# Association of Processed Meat Intake with Hypertension Risk in Hemodialysis Patients: A Cross-Sectional Study

**DOI:** 10.1371/journal.pone.0141917

**Published:** 2015-10-30

**Authors:** Pei-Yu Wu, Shwu-Huey Yang, Te-Chih Wong, Tzen-Wen Chen, His-Hsien Chen, Tso-Hsiao Chen, Yu-Tong Chen

**Affiliations:** 1 School of Nutrition and Health Science, Taipei Medical University, Taipei City, Taiwan; 2 Department of Nephrology, Taipei Medical University Hospital, Taipei City, Taiwan; 3 Department of Nephrology, Wan Fang Medical Center, Taipei City, Taiwan; The University of Manchester, UNITED KINGDOM

## Abstract

In this cross-sectional study, we hypothesized that hemodialysis patients consuming greater processed meat is associated with hypertension risk, which can be partly explained by the high sodium content in processed meat. From September 2013 to May 2014, one hundred and four patients requiring chronic hemodialysis treatment were recruited from hemodialysis centers. Data on systolic blood pressure and diastolic blood pressure before receiving dialysis, and 3-day dietary records of the recruited patients were collected. HD patients with systolic and diastolic blood pressures greater than140 mmHg and higher than 90 mmHg, respectively, were considered hypertension risk. Protein foods were divided into 4 categories: red meat, white meat, soybeans, and processed meat (e.g., sausage and ham). In a model adjusted for energy intake and hypertension history, additional servings of processed meats was positively associated to systolic blood pressure >140 mmHg (odds ratio [95% confidence interval]: 2.1 [1.0–4.3]), and diastolic blood pressure > 90 mmHg (odds ratio: 2.5 [1.2–5.5]). After adjustment for dietary sodium contents or body mass index (BMI), most associations were substantially attenuated and were no longer significant. In systolic blood pressure greater than140 mmHg, one serving per day of red meats (β = -1.22, *P* < .05) and white meats (β = -0. 75, *P* = .05) was associated with a reduced risk compared with one serving per day of processed meats. Similarly, compared with one serving per day of processed meat, a reduced risk of diastolic blood pressure higher than 90 mmHg was associated with one serving per day of red meat (β = -1. 59, *P* < .05), white meat (β = -0. 62, *P* < .05). Thus, in these hemodialysis patients, intake of processed meat is significantly positively associated with higher blood pressure risk, and both sodium contents in processed meat and BMI significantly contributes to this association.

## Introduction

The life expectancy of hemodialysis (HD) patients appears shorter than that of the general population with the same gender and age [1,2]. Cardiovascular disease (CVD) is considered the leading cause of death in HD patients, and reducing risk factors for CVD can decrease CVD mortality [1–3]. The results of the Nurses’ Health Study [4] and another survey recruited Swedish women (n = 345,760) [5] reveal that higher intake of processed meat is associated with increased risk of CVD. Processed meat mostly comprises pork and beef, which undergo treatment for improving their texture and flavor and increasing their preservation time [4]. Processed meat also contains high concentration of sodium and phosphorus [6,7]. Both HTN and hyperphosphatemia are risk factors for CVD in HD patients [8]. Greater sodium intake increases risk of hypertension (HTN) [9], and increase in phosphate intake elevates risk of hyperphosphatemia [10] in HD patients. However, most studies have investigated the effects of processed meat on hyperphosphatemia [10–12], and the association between processed meat and blood pressure in HD patients has been evaluate in a few study.

Inflammation is reported another major risk factor for CVD in HD patients [13]. In a cross-sectional study, higher sodium intake is positively associated with elevated concentration of high-sensitivity C-reactive protein (CRP) in the general population (n = 1597) [14]. In addition, a study on the chronic kidney disease animal model reveals that high sodium diet increases inflammation level [15]. Therefore, high sodium content in processed meat may elevate inflammation marker levels. In the Nurses’ Health Study, Ley et al. observe that greater processed meat intake is associated with increased serum CRP concentration [16]. Moreover, substituting one serving of red meat with that of white meat or plant protein substantially reduced the risks of inflammation, CVD incidence, and mortality in the Nurses’ Health Study [17,18]. Furthermore, the previous study reports the effects of processed meat intake on inflammation markers in the general population [19], but less study has focused on HD patients whose CVD risk is higher than the general population [1,2].

Therefore, in this study, we investigated the association between processed meat intake and two CVD risk factors in HD patients: HTN and inflammation marker levels. We also examined if substituting processed meat with another protein food could attenuate the positive association between processed meat and these two CVD risk factors.

## Materials and Methods

### Patients

**Fig 1:**
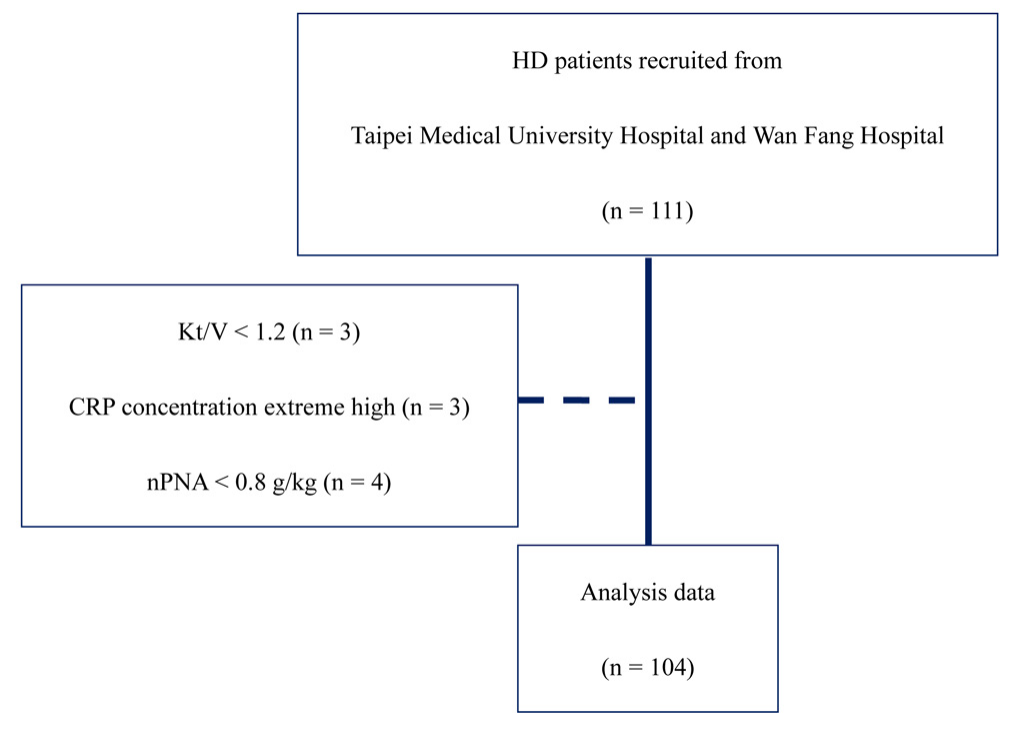
Flowchart of participants through the study.

One hundred and eleven chronic kidney disease patients from two HD centers affiliated with Taipei Medical University (Taipei, Taiwan) were recruited from September 2013 to June 2014. All participants regularly underwent HD treatment three times weekly with a dialysis time of 3.5–4 h per session for at least three consecutive months. Furthermore, all HD patients were aged >20 years and had no malignant tumors, cirrhosis, or acute infection, and were not hospitalized one month prior to recruitment. The excluding criteria were HD patients with an extremely high serum CRP concentration, Kt/V <1.2, or inadequate protein intake (normalized protein nitrogen appearance, nPNA: <0.8 [20]). Finally, one hundred and four patients were included in this study, and Fig 1 shows the recruitment flowchart. According to the FDAAA801 requirement, this objective study did not require registration in Clinicaltrial.gov. This study was conducted after approval by the Taipei Medical University Joint Institutional Review Board (no. 201302024). All patients signed consent forms before participation (S1 Appendix).

### Study procedures and main outcomes

In this cross-sectional and objective study, all data and information were collected in the same week. The medical chart was used to collect the data and information, excepting dietary history. The demographic data included gender, age, HD duration, medication and comorbidities, including HTN, diabetes, and CVD. Participants were considered with HTN history, if the diagnosis code from I10 to I15 in tenth revision of the international classification of diseases and health problems (ICD-10) was found in their medical chart. Manifestations of CVD included myocardial infarction (ICD 10 code I24.1), heart failure (ICD 10 code I50) and stroke (ICD 10 code I61-I64). In the anthropometric measurements, body mass index (BMI) was calculated from the dry body weight. The seated systolic blood pressure (SBP) and diastolic blood pressure (DBP) were measured using electronic BP monitors (Colin TP-8800 Series NIBP Monitor, Mexico or Terumo ES-P110, Japan). HD patients with a weekly average SBP >140 mmHg and DBP >90 mmHg were considered hypertension [21,22]. Besides, 64% of the patients (n = 57) had SBP >140 mmHg, 16% (n = 17) had DBP >90 mmHg, and 13% (n = 13) had both high SBP and DBP. However, only 10 patients (10%) took at least one kind of antihypertens(ion medication, and most of them used diuretic (n = 7) to control their blood pressure. Diuretic was deposited in the loop of Henle of renal tubules and interrupts sodium and chlorine reabsorption. Other antihypertensive medication used included angiotensin-converting-enzyme inhibitor (n = 6), and beta-adrenergic blocking agents (n = 4). All participants maintained their antihypertensive medication for at least 3 month before participation in this study. All serum samples were collected after fasting at least 8-h period and were then sent to the Department of Laboratory Medicine, Taipei Medical University Hospital for analysis. The serum concentration of CRP was measured using a particle-enhanced immunoturbidimetric assay (IMMULITE; Diagnostic Products Corp., Los Angeles, California), and the coefficient of variation was ≤5% at 0.20 mg/L of CRP. A previous study indicated that HD patients with a CRP concentration >3 mg/L (equal to 2.86 nM/L) had significantly higher mortality rate [23]. Therefore, serum CRP concentration >3 mg/L was defined as inflammation, which was observed in 45% (n = 47) of the HD patients in this study.

### Dietary intake

All patients were requested to white down a 3-day dietary record, which comprised details on one dialysis day and 2 nondialysis days (one each on a weekday and weekend). The process of collecting 3-day dietary record has been published [24]. In brief, trained dietitians taught the subjects, and provided the standardized instructions about how to complete the 3-day diet. Besides, all subjects had to meet the dietitians by face-to-face or telephone every day to confirm that they completed the dietary record. In the same week, the trained dietitian collected the 3-day dietary record, and another 24-h dietary recall was also collected face-to-face to correct the 3-day dietary records, particularly for fat, oil, and snacks [25]. All dietary records were evaluated by the same dietitian. The nutritional components were analyzed using nutrition analysis software (e-Kitchen) which was based on official publications of Taiwan food composition tables. Furthermore, the average dietary intake of each patient was used to calculate the result. nPNA was used as the indicator for dietary protein intake, and the following equation was applied: (mg/dL)/[25.8 + 1.15/(Kt/V) + 56.4/(Kt/V)] + 0.168 [26].

For assessing the reliability and validity of 3-day dietary records in this study, the Goldberg index and another 3-day dietary record of 77 participants were used [27,28]. The Goldberg index was the ratio of energy intake to the total energy expenditure (TEE) [27]. TEE was calculated by multiplying resting energy expenditure obtained using an indirect calorimeter (MedGem, Microlife USA, Dunedin, FL) by the physical activity level obtained using a structured questionnaire [29] in the same week for collection of the 3-day dietary record. Under-reporters were defined as those with the energy intake/TEE ratio of <0.81 for men and <0.79 for women. According to the cut-off points, 11.1% of female patients, 4.9% of male patients, and 7.8% of all patients were classified as under-reporters. This proportion was lower than that in a previous study [27,30]. The dry body weight of all 77 participants was the same in the two 3-dietary records within 1 month. Comparing the two 3-day dietary records, no statistical difference was observed in energy and dietary component intake (P > .05, S1 Table).

The protein foods were grouped according to the Nurses’ Health Study [16,17,31], Health Professionals Follow-Up Study [31], European Prospective Investigation into Cancer and Nutrition study [4], and Nutrition and Health Survey in Taiwan [6]. The protein foods were categorized into 4 groups: 1) processed meat (ham, sausage, hot dogs, pork floss, pork balls, and other instant foods); 2) red meat (fresh beef, pork, and lamb); 3) white meat (poultry, fish, seafood and eggs); and 4) soybeans (soybeans products, e.g. tofu and soy milk). Each serving of a protein source provided approximately 7 g of protein, which was almost equivalent to the protein content of an egg [32].

### Statistical analysis

The continuous variables were expressed as the mean ± standard error and mean (95% confidence intervals, CIs), and the categorical variables were expressed as the number of subjects (%). The normal distribution was assessed by the Kolmogorov–Smirnoff test. Gender-linked differences between groups was tested using Student's t-test for normally distributed data, and otherwise using the simple linear rank test. Categorical variable proportions were compared between groups by using the chi squared test. Furthermore, multiple robust regression models were used for analyzing the association between the different protein foods and blood pressure and serum CRP concentrations. Multiple logistic regression models were used to determine the odds ratio for hypertension and inflammation among different protein foods. Model 1 was adjusted for dietary energy, and Model 2 was additionally adjusted for gender, age, HTN medication (only for SBP and DBP), and HD duration (only for serum CRP). Because HTN history and HTN medication had collinearity in the regression model for hypertension, and HTN medication had higher predictive power. Therefore, this study did not adjust for HTN history. In addition, because the BMI [16] and sodium content in food [8] may modify the association between different protein foods and the CVD risk factors: HTN and inflammation, Model 2 was additionally adjusted for BMI, sodium contents, and their interaction with the protein foods. Phosphorus and fatty acids components in processed meat may also affect the association of processed meat and hypertension [33–35]. Besides, in this study, there was a slightly positive association between phosphorus and sodium contents in processed meat (r square = 0.03, *P* = 0.04). Therefore, both phosphorus and fatty acids components would be considered in the Model 2.

The effects of substituting one serving of processed meat with one serving of another protein foods was estimated by including both sources as continuous variables in the same multiple regression model [16,17]. The difference in the coefficients, variance and covariance between different protein foods was used to estimate the coefficients ± SEs and P values of the substitution effect [16]. The SAS 9.3 program (SAS Institute Inc., Cary, North Carolina) was used for performing statistical analysis. A 2-tailed p value of <0.05 was considered significant.

## Results

The demographic data of 104 participants was shown in Table 1. Their mean age was 62.3 ± 1.5 years old, and 48% (n = 50) of them were male participants. In this study, the median of HD duration was 6.5 years (range: 0.2–19.6 y). In comorbidity, percentage of fifty-two (n = 54) had HTN, and 49% (n = 51) had CVD. The mean SBP was 144.5 ± 2.4 mmHg, DBP was 76.6 ± 1.4 mmHg, and serum CRP level was 5.0 ± 0.8 mg/L. Except for the male participants with greater dietary energy and phosphate intake, no significant difference was observed between different gender groups.

**Table 1:** Descriptive data of all HD patients

	All patients (n = 104)	Male patients (n = 50)	Female patients (n = 54)
Age (y)	62.3 ± 1.5	63.9 ± 2.1	60.6 ± 2.1
Hemodialysis duration (y)	4.1 (0.2, 21.5)	4.1 (0.5, 21.5)	4.2 (0.2, 17.4)
Charlson comorbidity index	1.8 ± 0.1	1.7 ± 0.2	1.8 ± 0.2
Comorbidity (n)			
Diabetes	48 (46%)	26 (52%)	21 (39%)
Hypertension	54 (52%)	28 (55%)	26 (49%)
Cardiovascular disease	49 (47%)	19 (38%)	30 (56%)
SBP (mmHg)	144.5 ± 2.4	146.7 ± 3.7	142.1 ± 2.9
DBP (mmHg)	76.6 ± 1.4	78.8 ± 2.2	74.3 ± 1.7
C-reactive protein (mg/L)	5.0 ± 0.8	5.6 ± 1.2	4.5 ± 1.0
Serum phosphate (mg/dL)	5.3 ± 0.2	5.7 ± 0.3	5.1 ± 0.2
Kt/v	1.7 ± 0.0	1.6 ± 0.1	1.7 ± 0.1
Red meats (servings)	1.0 ± 0.1	1.1 ± 0.2	0.8 ± 0.2
Processed meats (servings)	1.3 ± 0.2	1.6 ± 0.3	1.1 ± 0.2
White meats (servings)	2.4 ± 0.2	2.4 ± 0.2	2.3 ± 0.2
Soybeans (servings)	0.5 ± 0.1	0.5 ± 0.1	0.6 ± 0.1
Energy intake (kcal/day)	1550.1 ± 56.4	1712.1 ± 79.1	1384.3 ± 72.7*
nPCR (g/kg/day)	1.2 ± 0.0	1.2 ± 0.1	1.2 ± 0.0
Dietary phosphoate (mg)	632.5 ± 32.9	760.0 ± 68.1	552.8 ± 30.8*

SBP: systolic blood pressure, DBP: diastolic blood pressure, nPCR: normalized protein catabolic rate.

* p < 0.05.

All patients consumed 1.3 ± 0.2 servings of processed meat, 1.0 ± 0.1 servings of red meat, 2.4 ± 0.2 servings of white meat, 0.5 ± 0.1 servings of soybeans (Table 1). The top 3 sources of processed meat were pork balls, ham, and pork floss. The sodium in processed meat was 1.6–45.7 times higher than red meats, 2.6–37.1 times higher than white meats, and 1.5–815.3 times higher than soybeans was found (Table 2). In this study, no linear association (*P* > 0.05) was observed between processed meats intake and serum CRP concentration, SBP, and DBP.

**Table 2:** Sodium and phosphorus content of the protein foods in HD patients

Protein foods	g/serving	Na (mg)/serving	P (mg)/serving	P (mg)/ protein (g)
Red meats				
Pork belly	50	18.0	64.0	8.8
Lean pork	35	13.7	78.1	10.8
Lean beef	35	27.9	92.3	12.6
Pork Loin	35	10.7	11.6	1.7
White meats				
Fish	35	11.2–28.0	62.7–71.2	8.9–11.2
Chicken	35	13.2	21.6	4.5
Egg	55	74.3	101.8	15.3
Egg white	70	90.3	4.2	0.7
Soybeans				
Soy milk	260	112.0	140.0	13.0
Dried tofu	45	155.8	101.9	15.1
Tofu	80/140	0.8	88.8	13.1
Fried tofu	55	0.6	119.9	17.2
Processed meats				
Pork ball	40	232.8	68.4	10.4
Ham	45	489.2	132.8	18.8
Pork floss	20	293.4	56.2	8.5
Mix food				
Dumpling	45	141.3	27.5	7.8
Pork bun	40	96.4	125.6	44.2

Na: sodium, P: phosphorus.

SBP >140 mmHg and DBP >90 mmHg were considered as HTN in this study. After adjustment for gender, age, HTN medication, and dietary energy intake, each additional serving of processed meat positively associated with the risk of HTN (Table 3). The odds ratio for SBP >140 mmHg was 2.10 (95% CI, 1.03 to 4.30) and odds ratio for DBP >90 mmHg was 2.51 (95% CI, 1.15 to 5.47). Furthermore, an increasing intake of 1 serving size of red meat was negatively associated with the risk of HTN, and the odds ratio for SBP >140 mmHg was 0.48 (95% CI: 0. 27, 0. 88) and DBP >90 mmHg was 0. 38 (95% CI: 0. 20, 0. 74). After further adjustment of sodium contents or BMI, these associations with SBP >140 mmHg and DBP >90 mmHg attenuated and were no longer significant. Both of white meats and soybeans were not significantly associated with blood pressure of HD patients. Patients with a serum CRP concentration >3 mg/L were considered to have inflammation, and no association was observed between inflammation and the 4 protein foods.

**Table 3:** Odds ratio of one serving per day of protein foods intake and CVD risk factors^1^.

	CRP >3 mg/L	SBP >140 mmHg	DBP >90 mmHg
	**Odds ratio (95% CI)**		
Red meats			
Model 1	0.85 (0.62, 1.15)	0.46 (0.32, 0.66)	0.33 (0.22, 0.52)
Model 2	0.87 (0.64, 1.19)	0.48 (0.27, 0.88)	0.38 (0.20, 0.74)
Model 2 + BMI	1.00 (0.08, 11.98)	1.28 (0.80, 2.03)	0.74 (0.38, 1.45)
Model 2 + sodium	0.63 (0.36, 1.12)	1.21 (0.77, 1.92)	0.72 (0.38, 1.37)
White meats			
Model 1	0.96 (0.76, 1.22)	0.79 (0.62, 1.01)	0.92 (0.72, 1.16)
Model 2	0.98 (0.76, 1.25)	0.81 (0.56, 1.16)	0.83 (0.58, 1.19)
Model 2 + BMI	1.22 (0.14, 10.50)	0.93 (0.55, 1.59)	1.48 (0.70, 3.12)
Model 2 + sodium	0.76 (0.48, 1.22)	0.92 (0.54, 1.56)	1.32 (0.65, 2.67)
Soybeans products			
Model 1	1.70 (0.92, 3.13)	1.12 (0.73, 1.70)	1.01 (0.67, 1.53)
Model 2	1.68 (0.90, 3.13)	1.00 (0.62, 1.63)	0.73 (0.35, 1.51)
Model 2 + BMI	1.18 (0.01, 2.34)	1.07 (0.64, 1.76)	0.66 (0.31, 1.44)
Model 2 + sodium	0.85 (0.20, 3.51)	0.97 (0.59, 1.62)	0.62 (0.29, 1.32)
Processed meats			
Model 1	0.89 (0.71, 1.12)	1.69 (1.30, 2.21)	1.84 (1.33, 2.56)
Model 2	0.86 (0.67, 1.10)	2.10 (1.03, 4.30)	2.51 (1.15, 5.47)
Model 2 + BMI	0.20 (0.04, 0.96)	1.21 (0.82, 1.76)	1.21 (0.74, 1.97)
Model 2 + sodium	0.69 (0.39, 1.22)	1.17 (0.80, 1.71)	1.16 (0.72, 1.86)

Values presents as odds ratio (95% CI).

Model 1: adjusted for dietary energy. Model 2 for blood pressure: adjusted for gender, age, HTN medication, and dietary energy, Model 2 for inflammation status: adjusted for gender, age, hemodialysis duration and dietary energy. Model 2 + BMI included adjusting for the interaction between BMI and protein foods. Model 2 + sodium included adjusting for the interaction between sodium contents and protein foods.

^1^The unit for OR was 1 serving size of protein foods.

The substitution effects were estimated according to exchanging one serving of processed meat intake with another protein food containing equal protein. Substituted one serving of processed meat with one serving of red meat or white meat was negatively associated with risk of SBP >140 mmHg and DBP >90 mmHg in HD patients (Table 4). These effects became attenuated to non-significance after additional adjustment of models for sodium contents or BMI. Moreover, after further adjusted for the saturated fatty acids (percentage of dietary energy), the ratio of unsaturated fatty acids to saturated fatty acids, serum phosphate, or the interaction between phosphorus and sodium contents in processed meat, substituting processed meat with other protein foods remained significantly associated with SBP >140 mmHg and DBP >90 mmHg (data did not shown).

**Table 4:** Changes in risk of inflammation and hypertension corresponding to substitution of one serving of processed meat with that of other protein foods

	CRP > 3 mg/L	SBP > 140 mmHg	DBP > 90 mmHg
	**β ± SE**		
Model 2			
Red meats	-0.01 ± 0.18	-1.22 ± 0.42*	-1.59 ± 0.65*
White meats	0.13 ± 0.18	-0.75 ± 0.38^†^	-0.62 ± 0.19*
Soybeans products	0.62 ± 0.34	-0.14 ± 0.32	-0.61 ± 0.46
Model 2 + BMI			
Red meats	1.48 ± 0.82	-0.36 ± 0.56	-0.41 ± 0.39
White meats	1.55 ± 0.82	-0.22 ± 0.32	-0.18 ± 0.64
Soybeans products	1.95 ± 0.87*	-0.62 ± 0.72	-0.60 ± 0.47
Model 2 + sodium			
Red meats	-0.01 ± 0.18	0.05 ± 0.28	-0.38 ± 0.37
White meats	0.13 ± 0.18	-0.21 ± 0.32	0.13 ± 0.41
Soybeans products	0.62 ± 0.34	-0.19 ± 0.33	-0.64 ± 0.47

* p < 0.05

† p = 0.05.

The equation included processed meats and alternative protein foods at the same time, and Model 2 for blood pressure: adjusted for gender, age, HTN medication and dietary energy, Model 2 for inflammation status: adjusted for gender, age, hemodialysis duration and dietary energy. Model 2 + BMI included adjusting for the interaction between BMI and processed meat. Model 2 + sodium included adjusting for the interaction between sodium contents and protein foods.

## Discussion

For HD patients, SBP >140 mmHg was associated with an elevated risk of CVD events and mortality [36]. In this study, greater intake of processed meat was positively associated with the risk of HTN. After adjustment for sodium contents or BMI, the association with HTN was attenuated, which meant both sodium contents and BMI accounted for significant proportion of this association. Substituting a serving of per day processed meat intake with that of red meat and white meat was negatively associated with the HTN risk.

This study revealed that greater intake of processed meat, but not unprocessed red meat, was associated with higher HTN risk in HD patients. This result was consistent with a previous 15-year prospective study conducted among a population of 44,616 disease-free French women, in whom a higher processed meat intake was associated with elevated HTN risk [37]. Compared with French women who consumed less than 1 serving per week of processed meat, women who consumed ≥5 servings per week had a 17% higher rate of HTN. Furthermore, the Nurses’ Health Study reported that a higher intake of processed meat was significantly associated with increased CVD mortality [17]. An 11.8-year follow-up study of a cohort of Swedish men revealed a positive association between the intake of processed meat and incidence of heart failure [38]. CVD was the primary cause of death in HD patients [39], and HTN was a major risk factor for CVD in both general population and HD patients [40,41]. Future studies were necessary for clarifying the association between processed meat intake and CVD mortality in HD patients.

In 2000, the National Heart, Lung, and Blood Institute circulated a warning that higher sodium intake would increase HTN risk [42]. In accordance, the results of this study revealed that the association between processed meat and HTN risk was significantly attenuated after additional adjustment for sodium intake. Processed meat was the major source of sodium [43]. According to the Nutrition and Health Survey in Taiwan, the average sodium intake is 4070 mg/day, and processed meat contributes to 43% of the total sodium intake [6]. In this study, the amount of sodium from processed meat was 1.5–815.3 times higher than other protein foods. For instance, one serving of ham contains 489.2 mg of sodium, and one serving of pork belly contains only 18.0 mg of sodium. By contrast, the results of this study suggested that the effects of substituting processed meat with red and white meat were not significant after adjustment for dietary sodium contents. This was consistent with a previous study stating that reducing the intake of processed meat lowers sodium intake [44].

Studies have suggested that dialysis patients should avoid excess sodium and alcohol to control their blood pressure [45,46]. In this study, all subjects do not drink any alcohol. In addition, the Dietary Approaches to Stop Hypertension (DASH) eating pattern is suggested for lowering blood pressure in the general population [47]. The DASH diet is rich in fruits, vegetables, whole grains and low-fat dairy products and reduced in total and saturated fat, cholesterol, and sugar-sweetened products. However, the DASH diet is rich in potassium and phosphorus, which increase the risks of cardiac arrhythmias, hyperphosphatemia, and CVD mortality in dialysis patients [42]. Therefore, the DASH eating pattern is not recommended for dialysis patients [42]. In addition, patients with hemodialysis treatment have lower intake of fruits and vegetables than general population [48].

In this study, BMI was another significant factor in the association between processed meat and HTN. This result was consistence with that of the Nurses’ Health Study [16]. The possible reason may be that higher sodium content in processed meat result in elevated dry body weight in HD patients [49], and BMI was positively associated with HTN risk in hemodialysis patients [50,51]. In general population, different protein foods intake may affect the BMI [52]. In the meta-analysis study, results showed that processed meat related to higher obesity risk [53]. However, this was a cross-sectional study, and it was difficult to explain the causality of the association between protein foods, hypertension and BMI. Therefore, further long-term intervention studies are needed to find out the mechanisms in HD patients.

Previous studies have reported that higher intake of processed meat elevated the concentration of serum CRP in the general population [5,16]. Furthermore, greater sodium intake may not only increase the HTN risk but also aggravate inflammation [14]. However, we did not observe similar associations in this study. One possible reason was the recruited HD patients with mild inflammation. The serum CRP concentration was 0.5 ± 0.1 mg/L (4.8 ± 0.7 nM/L), and lower than the concentrations reported in 29,842 HD patients (in males, 117 ± 215 nM/L and in females, 105 ± 205 nM/L) from 12 countries in the Dialysis Outcomes and Practice Patterns Study (DOPPS) [54]. Moreover, in this study, only 33% (n = 34) and 13% (n = 14) had serum CRP concentrations >5 mg/L and >10 mg/L, respectively. By contrast, in previous studies, 40.6%–65% and 32%–53% of patients had serum CRP concentrations >5 mg/L and >10 mg/L, respectively [23]. Moreover, all HD patients in this study routinely took folate and vitamin B complex supplements, which may contribute to the null effect. In a 3-month intervention study, folate and vitamin B complex supplements significantly reduced the serum level of CRP in HD patients [55].

Although the interaction between phosphorus and sodium in processed meats was found in this study, the association between processed meat and HTN was modified by neither dietary phosphorus nor the interaction between phosphorus and sodium contents in processed meat. These findings were inconsistent with those of a previous study [9], and some reasons may explain the result. At first, the course of processed meats production varied with the area, and some processed meats did not contain phosphorus [11,56]. In this study, the major sources of processed meats did not have higher phosphorus content than red meats. Table 2 listed up to 70% of all processed meat consumed by the patients. The phosphorus content ranged from 27.5 (dumplings) to 132.8 (ham) mg, and the ratio of phosphorus content to protein content was 1.7:18.8 mg/g. Moreover, the phosphorus content in major sources of red meat ranged from 11.6 (pork loin) to 92.3 (lean beef) mg, and the ratio of phosphorus content to protein content was 1.7:12.6 mg/g. At second, lack of the relation between hyperphosphatemia and hypertension in HD patients. In chronic kidney disease patients, increased processed meat intake contributed to hyperphosphatemia [9], and hyperphosphatemia resulted in calcifications in the coronary arteries and higher CVD mortality [31,57,58]. Coronary artery calcification was associated with the development of hypertension in general population [35]. However, in chronic kidney disease patients with dialysis treatment, it seemed difficult to find the relation between hypertension and coronary artery calcifications [59–61].

The Kidney Disease Outcomes Quality Initiative recommended that HD patients consume adequate dietary protein for preventing malnutrition and increasing their survival rate [21]. The results in this study reported when protein intake was sufficient, different types of protein foods may affect the risk factors for CVD (e.g., hypertension) and mortality. The terms “hemodialysis,” “meats,” “inflammation,” and/or “blood pressure” were searched on PubMed and Google Scholar, and less than five articles were found. This search result implied that only few studies have focused on the association between different protein foods and CVD risk factors in HD patients, despite CVD being the major cause of death in HD patients. As per our review of relevant literatures, this study is the first to report that a higher intake of processed meat was positively associated with the risk of CVD in HD patients. These results could be considered evidence for future studies on clinical nutrition and encourage HD patients not only to consume an adequate quantity of protein but also to increase their intake of unprocessed red and white meat to reduce CVD risk.

This study had several limitations. First, the sample size was small. However, the demographic data of the HD patients in this study was consistent with those reported by previous large studies, including a study conducted at 25 HD centers in Taiwan [62], the Taiwan Renal Registry Data System [2], the United States Renal Data System [63], and the DOPPS [54]. Furthermore, the average age of the patients was 60.1–66.6 years, approximately 50% of the patients were male, and the pre-HD SBP was 145.9 ± 23.2 mmHg. Medical history review revealed that 36.2%–44.4% of the patients had diabetes, 71.1%–79.6% had HTN, and at least 20.35% had CVD. Although the prevalence of HTN was lower in this study than in previous studies, the results were independent of the HTN medication. Moreover, the association between the intake of processed meat and risk of SBP >140 mmHg and DBP >90 mmHg was significant. Second, selection bias existed during enrollment, and few patients took HTN medication, and the average serum CRP concentration was lower than that reported by a previous study [54]. Therefore, HD patients in this study may be healthier than those in a previous study [64]. Although the association between protein foods and blood pressure was independent of HTN medication, the future study with more dialysis patients using HTN medication is needed. Third, because this was a cross-sectional study, it was difficult to determine causal relationships. However, in Nurses’ Health Study [17], greater intake of processed meats is associated with higher risk of coronary heart disease, and substituting processed meats with white meats was associated with reduced risk of coronary heart disease. HTN is one major predictor of coronary heart disease [65]. Fourth, although a dietary record over multiple days was used to evaluate the validity of other dietary assessment tool [66,67], dietary records may underestimate the actual food intake [68]. However, the under-reported bias was lower in this study [27,30], more than 7% of participants under reported their dietary intake. Fifth, this study did not include all confounding factors for pre-HD blood pressure in HD patients, such as the concentration of sodium in the dialysis solution [69]. However, the results regarding factors influencing pre-HD blood pressure were still inconsistent [70], and future interventional studies are required to assess the effects of processed meat on this factor in HD patients.

In conclusion, greater intake of processed meat by HD patients is positively associated with HTN risk, and this observation may be explained by the high sodium content in processed meat and BMI. Moreover, substituting one serving of processed meat with that of unprocessed red and white meat is negatively associated with HTN risk.

## Supporting Information

S1 Appendix The certification of Taipei Medical University Joint Institutional Review Board (DOCX)

S2 Appendix STROBE Statement—checklist of items that should be included in reports of observational studies (DOCX)

S1 Table Comparison of mean daily intake between two 3-day dietary records (Mean ± SE) (DOCX)
