# Supplementary material for: Association of Processed Meat Intake with Hypertension Risk in Hemodialysis Patients: A Cross-Sectional Study
Source: PLoS One. 2015 Oct 30;10(10):e0141917. doi: 10.1371/journal.pone.0141917 (PMC4627724; doi:10.1371/journal.pone.0141917)
Supplement: S2 Appendix — (DOCX) [file pone.0141917.s002.docx]

S2 Appendix. STROBE Statement—checklist of items that should be included in reports of observational studies

|  | Item No. | Recommendation | Page  No. | Relevant text from manuscript |
| --- | --- | --- | --- | --- |
| **Title and abstract** | 1 | (*a*) Indicate the study’s design with a commonly used term in the title or the abstract | 1 | More processed meats intake is related to higher hypertension risk in maintenance hemodialysis patients: a cross-sectional study |
|  |  | (*b*) Provide in the abstract an informative and balanced summary of what was done and what was found | 3 | Therefore, in these hemodialysis patients, greater processed meats intake is associated to risk of hypertension, and sodium intake significantly contributes to the association. |
| Introduction | | | |  |
| Background/rationale | 2 | Explain the scientific background and rationale for the investigation being reported | 4 | The results of Nurses’ Health Study, and another survey recruiting Swedish women (n = 345 760)4 indicated that higher intake of processed meats is associated with increased risk of CVD. Processed meats are mostly from pork and beef which under treatment to improve their texture, flavor, and longer preservation. Processed meats also contain high sodium and phosphorus. Greater sodium intake increases the risks of hypertension (HTN) |
| Objectives | 3 | State specific objectives, including any prespecified hypotheses | 5 | Therefore, the association between the processed meats intake and hypertension (HTN) and the markers for inflammation in HD patients was investigated. Besides, we also hypothesis that substitute processed meats with another protein food would be associated with lower risk of HTN and inflammation. |
| Methods | | | |  |
| Study design | 4 | Present key elements of study design early in the paper | 6 | This was a cross-sectional study. |
| Setting | 5 | Describe the setting, locations, and relevant dates, including periods of recruitment, exposure, follow-up, and data collection | 6 | All participants were recruited from 2 HD centers affiliated with the Taipei Medical University (Taipei, Taiwan) from September 2013 to June 2014……. All data and information were collected in the same week. |
| Participants | 6 | (*a*) *Cohort study*—Give the eligibility criteria, and the sources and methods of selection of participants. Describe methods of follow-up  *Case-control study*—Give the eligibility criteria, and the sources and methods of case ascertainment and control selection. Give the rationale for the choice of cases and controls  *Cross-sectional study*—Give the eligibility criteria, and the sources and methods of selection of participants | 5 | One hundred and eleven of participants receiving regular HD treatment 3 times per week for at least 3 consecutive months were recruited in this study. All participants were older than 20 years and without malignant tumors, cirrhosis, acute infection, HD session duration < 3.5 h or hospitalized one month prior to the recruitment. We also excluded HD patients with an extreme high level of serum CRP, Kt/V <1.2 or inadequate protein intake (the normalized protein nitrogen appearance [nPNA] <0.8) |
|  |  | (*b*) *Cohort study*—For matched studies, give matching criteria and number of exposed and unexposed  *Case-control study*—For matched studies, give matching criteria and the number of controls per case | This is neither cohort study nor case-control study | This is neither cohort study nor case-control study |
| Variables | 7 | Clearly define all outcomes, exposures, predictors, potential confounders, and effect modifiers. Give diagnostic criteria, if applicable | 7, 8 | Participants with weekly averaged SBP >140 mmHg and DBP >90 mmHg were considered as having HTN.  ……the protein food included 4 group: 1) processed meats, included ham, sausage, hot dogs, pork floss, pork balls, and other instant foods; 2) red meats, included fresh beef, pork, and lamb; 3) white meats included poultry, fish and seafood, and eggs; 4) soybeans, included soybeans product, for example tofu and soy milk. Each serving of protein food provided ~7 g of protein, which is almost equivalent an egg |
| Data sources/ measurement | 8* | For each variable of interest, give sources of data and details of methods of assessment (measurement). Describe comparability of assessment methods if there is more than one group | 6, 8 | The seated systolic blood pressure (SBP) and diastolic blood pressure (DBP) was measured by electronic BP monitors (Colin TP-8800 Series NIBP Monitor, Mexico or TERUMO ES-P110, Japan).  ……The participants were requested to write a 3-day dietary record, and included one dialysis day, one nondialysis day, and one weekend nondialysisday.24 Another 24-h dietary recall was collected to ensure the contents of 3-day dietary recorded accurate. |
| Bias | 9 | Describe any efforts to address potential sources of bias | 22 | the selection bias existed during the enrollment. Less participants with HTN history and the average serum CRP concentration was relative low. |
| Study size | 10 | Explain how the study size was arrived at | 21 | The sample size is limit. However, the demographic data of HD patients in this study was consistent with previous large studies, including the study conducted at 25 HD centers in Taiwan, the Taiwan Renal Registry Data System, the United States Renal Data System, and the Dialysis Outcomes and Practice Patterns Study (DOPPS), |

Continued on next page

| Quantitative variables | 11 | Explain how quantitative variables were handled in the analyses. If applicable, describe which groupings were chosen and why | 6, 8 | The demographic data included gender, age, HD duration, and comorbidities, including HTN, diabetes, and CVD. The anthropometric measurements included the dry body weight and calculated body mass index (BMI). The seated systolic blood pressure (SBP) and diastolic blood pressure (DBP) was measured by electronic BP monitors (Colin TP-8800 Series NIBP Monitor, Mexico or TERUMO ES-P110, Japan).  ……In addition, the nPNA was used as the indicator for dietary protein intake, and the equation was as follows: (mg/dL)/[25.8+(1.15/(Kt/V)+56.4/(Kt/V)]+0.168.  ……the protein food included 4 group: 1) processed meats, included ham, sausage, hot dogs, pork floss, pork balls, and other instant foods; 2) red meats, included fresh beef, pork, and lamb; 3) white meats included poultry, fish and seafood, and eggs; 4) soybeans, included soybeans product, for example tofu and soy milk. Each serving of protein food provided ~7 g of protein, which is almost equivalent an egg. |
| --- | --- | --- | --- | --- |
| Statistical methods | 12 | (*a*) Describe all statistical methods, including those used to control for confounding | 9 | Normal distribution was assessed by the Kolmogorov-Smirnoff test. Differences between gender groups was tested using Student's t-test for normally distributed data, and otherwise with the Npar1way Wilcoxon. Categorical variable proportions are compared between groups with chi-square test. Multiple robust regression models were used to analyze relation between the different protein food and blood pressure and serum level of CRP. Multiple logistic regression models were calculated to determine the odds ratio (OR) for hypertension and inflammation among different protein. Model 1 was adjusted for dietary energy, and Model 2 was additional adjusted for gender, age, HTN history (only for SBP and DBP) or HD period (only for serum CRP). Because BMI16 and the sodium content in food8 may modify the correlation between different protein foods and hypertension and inflammation. Model 2 with additionally adjusted for BMI or dietary sodium intake were also used.  The effect of substituting 1 serving of processed meats with 1 serving of another protein food was estimated by including both as continuous variables in the same multiple regression model with adjusted for Model 2 and Model 2 with additionally adjusted for dietary sodium intake. |
|  |  | (*b*) Describe any methods used to examine subgroups and interactions | 9 | Multiple logistic regression models were calculated to determine the odds ratio (OR) for hypertension and inflammation among different protein. Model 1 was adjusted for dietary energy, and Model 2 was additional adjusted for gender, age, HTN history (only for SBP and DBP) or HD period (only for serum CRP). Because BMI16 and the sodium content in food8 may modify the correlation between different protein foods and hypertension and inflammation. Model 2 with additionally adjusted for BMI or dietary sodium intake were also used. The effect of substituting 1 serving of processed meats with 1 serving of another protein food was estimated by including both as continuous variables in the same multiple regression model with adjusted for Model 2 and Model 2 with additionally adjusted for dietary sodium intake. |
|  |  | (*c*) Explain how missing data were addressed | No missing data in this study | No missing data in this study |
|  |  | (*d*) *Cohort study*—If applicable, explain how loss to follow-up was addressed  *Case-control study*—If applicable, explain how matching of cases and controls was addressed  *Cross-sectional study*—If applicable, describe analytical methods taking account of sampling strategy | No analytical methods taking account of sampling strategy | No analytical methods taking account of sampling strategy |
|  |  | (*e*) Describe any sensitivity analyses | 9 | Multiple robust regression models were used to analyze relation between the different protein food and blood pressure and serum level of CRP. Multiple logistic regression models were calculated to determine the odds ratio (OR) for hypertension and inflammation among different protein. Model 1 was adjusted for dietary energy, and Model 2 was additional adjusted for gender, age, HTN history (only for SBP and DBP) or HD period (only for serum CRP). Because BMI16 and the sodium content in food8 may modify the correlation between different protein foods and hypertension and inflammation. Model 2 with additionally adjusted for BMI or dietary sodium intake were also used.  The effect of substituting 1 serving of processed meats with 1 serving of another protein food was estimated by including both as continuous variables in the same multiple regression model with adjusted for Model 2 and Model 2 with additionally adjusted for dietary sodium intake. |
| Results | | | | |
| Participants | 13* | (a) Report numbers of individuals at each stage of study—eg numbers potentially eligible, examined for eligibility, confirmed eligible, included in the study, completing follow-up, and analysed | 10 | The demographic data of 104 participants was shown in Table 1 |
|  |  | (b) Give reasons for non-participation at each stage | 6 | Fig 1. |
|  |  | (c) Consider use of a flow diagram | 6 | Fig 1. |
| Descriptive data | 14* | (a) Give characteristics of study participants (eg demographic, clinical, social) and information on exposures and potential confounders | 10 | Their mean age was 62.3 ± 1.5 years old, and 48% (n = 50) of them were male participants. In this study, the median of HD duration was 6.5 years (range: 0.2-19.6 y). In comorbidity, percentage of fifty-two (n = 54) had HTN, and 49% (n = 51) had CVD. The mean SBP was 144.5 ± 2.4 mmHg, DBP was 76.6 ± 1.4 mmHg, and serum CRP level was 5 ± 0.8 mg/L. |
|  |  | (b) Indicate number of participants with missing data for each variable of interest | No missing data in this study | No missing data in this study |
|  |  | (c) *Cohort study*—Summarise follow-up time (eg, average and total amount) | This is a cross-sectional study | This is a cross-sectional study |
| Outcome data | 15* | *Cohort study*—Report numbers of outcome events or summary measures over time |  |  |
|  |  | *Case-control study—*Report numbers in each exposure category, or summary measures of exposure |  |  |
|  |  | *Cross-sectional study—*Report numbers of outcome events or summary measures | 10 | Table 1 |
| Main results | 16 | (*a*) Give unadjusted estimates and, if applicable, confounder-adjusted estimates and their precision (eg, 95% confidence interval). Make clear which confounders were adjusted for and why they were included | 15, 16 | Table 3, Table 4 |
|  |  | (*b*) Report category boundaries when continuous variables were categorized | 13 | SBP >140 mmHg and DBP >90 mmHg were defined as HTN in this study, and each additional serving of processed meats increased the risk of HTN after adjustment for gender, age, HTN history, and dietary energy intake (Table 3). |
|  |  | (*c*) If relevant, consider translating estimates of relative risk into absolute risk for a meaningful time period | This is a cross-sectional study | This is a cross-sectional study |

Continued on next page

| Other analyses | 17 | Report other analyses done—eg analyses of subgroups and interactions, and sensitivity analyses | 15, 16 | Table 3, Table 4 |
| --- | --- | --- | --- | --- |
| Discussion | | | | |
| Key results | 18 | Summarise key results with reference to study objectives | 18 | For HD patients, SBP >140 mmHg was associated with elevated CVD events and mortality.28 In this study, greater intake of processed meat was associated with higher risk of SBP >140 mmHg and DBP >90 mmHg. Both of SBP >140 mmHg and DBP >90 mmHg were considered HTN in this study.21, 22 After addition adjustment for dietary sodium intake, the association with HTN was attenuated. Dietary sodium intake accounted for a statistically significant proportion of the association. Substitution of a serving of processed meat intake with red meat and white meat was associated with lower risk of HTN. |
| Limitations | 19 | Discuss limitations of the study, taking into account sources of potential bias or imprecision. Discuss both direction and magnitude of any potential bias | 22 | There were three limitations in this study though. First, the sample size is limit. However, the demographic data of HD patients in this study was consistent with previous large studies, including the study conducted at 25 HD centers in Taiwan,41 the Taiwan Renal Registry Data System,2 the United States Renal Data System,42 and the Dialysis Outcomes and Practice Patterns Study (DOPPS)38, the average age was between 60.1 to 66.6 year; the male participants accounted for nearly 50% of the participants; in medical history, 36.2%–44.4% with diabetes, 71.1%–79.6% with HTN, and at least 20.35% with CVD; the pre-HD SBP was 145.9 ± 23.2 mmHg. Although HD patients in this study had lower prevalence of HTN history than previous studies, the results in this study were independence of HTN history. In addition, the association between intake in processed meats and risk of SBP >140 mmHg and of DBP >90 mmHg was statistically significant. Second, the selection bias existed during the enrollment. Less participants with HTN history and the average serum CRP concentration was relative low.38 Therefore, participants in this study may be healthier than other HD patients.43 However, the association between protein foods and blood pressure was independent of HTN history. Third, the cross-sectional study design could not determine causal relationships. However, the results were consistent with those of the Nurses’ Health Study, 17 such as greater intake of processed meats was associated with higher risk of coronary heart disease; substituting processed meats with white meats was associated with reduced risk of coronary heart disease.17 Third, this study did not include all confounding factors for pre-HD blood pressure in HD patients, such as the concentration of sodium in the dialysis solution.44 However, the results of influencing factors of pre-HD blood pressure were considered inconsistent,45 and future interventional studies are planned to document the effect of processed meats on pre-HD blood pressure in HD patients. |
| Interpretation | 20 | Give a cautious overall interpretation of results considering objectives, limitations, multiplicity of analyses, results from similar studies, and other relevant evidence | 18-22 | This study found out that greater intake of processed meats but not unprocessed red meat intake was associated with higher HTN risk in HD patients, which is consistent with the previous study in the general population.29 In this 15-year prospective study among 44,616 disease-free French women, higher processed meat intake was positively related to incident HTN. Comparing with the less than one serving per week of processed meat intake, French women who consumed ≥ 5 servings/week had a 17% higher rate of hypertension. Processed meat intake had similar association with CVD incidence and mortality in the general population.17, 30 The Nurses’ Health Study have document that higher intake of processed meats is significantly related to increased CVD mortality.17 After 11.8 years of follow-up, the Cohort of Sweden Men study also found out the positive association between processed meat intake and incidence of heart failure. 30 CVD was the primary cause of death for HD patients,31 and HTN was one of the primary risk factors for CVD both in general population and HD patients.32,33 Future studies were necessary to document the association between processed meat intake and CVD mortality in HD patients.  Since 2000, the National Heart, Lung, and Blood Institute have warned that an increase in sodium intake increase the risk of HTN.34 The results of this study showed that the association between processed meats and risk of SBP >140 mmHg and DBP >90 mmHg was attenuated to not significantly after additional adjustment for sodium intake. Processed meats were the major sources of sodium intake.35 According to the Nutrition and Health Survey in Taiwan, average sodium intake was 4070 mg/day, and 43% of sodium intake came from processed meats.6 The sodium consumed by the participants from processed meats was 1.5–815.3 times more than the sodium content in other proteins. For example, a serving of ham contains 489.2 mg of sodium, whereas a serving of lean pork contained only 10.7 mg of sodium. On the other hand, the results of this study suggested that the substitution effect of processed meats with red meats and whites also became no longer significant in this study after adjusted for dietary sodium intake. This was consistent with previous study that reduced intake of processed meats lowers the sodium intake.36  No observation in this study found that BMI significantly modify the association between processed meats and HTN. This result was inconsistent with the Nurses’ Health Study, which considered that BMI significantly affect the association between processed meats and HTN.16 The different study population may contribute to inconsistent results between this study and previous study. The participants in this study were HD patients, while those in the Nurses’ Health Study were general population. Obesity was a well-known risk factor for CVD in the general population, but it is reported that obesity HD patients do not have a significantly increased CVD incidence. On the contrary, obesity HD patients have significantly reduced risk of deaths from CVD.37  Previous studies report that greater intake of processed meats elevates the serum CRP concentration in general population.4,16 Furthermore, higher sodium intake may not only increased the risk of HTN, but also aggravate inflammation.14 However, this study did not find out similar association. This is possibly because of the recruited HD patients with mild inflammation than those participating in the previous studies. The serum CRP concentration of the participants in this study was 0.5 ± 0.1 mg/L (4.8 ± 0.7 nM/L), which was lower than 29 842 HD patients from 12 countries in DOPPS (for male patients was 117 ± 215 nM/L and for females was 105 ± 205 nM/L).38 In addition, among the participants in this study, only 33% (n = 34) had a serum CRP concentration >5 mg/L, and 13% (n = 14) had a serum CRP concentration >10 mg/L. While in the previous studies, 40.6%–65% of the patients had serum CRP concentration >5 mg/L, and 32%–53% had serum CRP concentration >10 mg/L.23 All participants in this study have routine use of folate and vitamin B complex supplement which may contribute to the null effect. In the 3-month intervention study, folate and vitamin B complex supplement significantly reduce serum level of CRP in HD patients. 39  The top sources of processed meats did not contain higher phosphate in this study. Table 2 lists the up to 70% of processed meats consumed by the participants. The phosphorus content was from 27.5 (dumpling) to 132.8 (ham) mg, and the ratio between phosphorus and protein was 1.7 to 18.8 mg/g. The phosphorus in top sources of red meats was 11.6 (pork loin) to 92.3 (lean beef) mg, and the ratio between phosphorus and protein was 1.7 (pork loin)–12.6 (lean beef) mg/g. However, the results were inconsistent with the results of previous studies.9 This may be because the types of the top source of processed meats were various in different areas, and some processed meats do no add phosphorus.11,40  The Kidney Disease Outcomes Quality Initiative recommended HD patients with adequate dietary protein intake to prevent malnutrition and to increase survival rate.21 When protein intake is sufficient, different types of protein foods may affect the risk factors for CVD (e.g., hypertension) and mortality. The terms “hemodialysis”, “meats”, “inflammation”, and/or “blood-pressure” were searched on the PubMed and on Google, but only less than 5 articles were found. This implies that few studies have focused on the association between the different types of protein foods and the risk factors for CVD in HD patients, though CVD is the major cause of HD patients. As we know, this study was the first study to report that greater processed meats intake was associated with increased risk factors of CVD for HD patients. These results could be applied as evidence base for future clinical nutrition education, and encourage HD patients to consume not only adequate quantity of protein, but also more unprocessed red meat and white meat for lower CVD risk. |
| Generalisability | 21 | Discuss the generalisability (external validity) of the study results | 22 | the demographic data of HD patients in this study was consistent with previous large studies, including the study conducted at 25 HD centers in Taiwan, the Taiwan Renal Registry Data System, the United States Renal Data System, and the Dialysis Outcomes and Practice Patterns Study (DOPPS) |
| Other information | |  | | |
| Funding | 22 | Give the source of funding and the role of the funders for the present study and, if applicable, for the original study on which the present article is based | The source of funding is not presenting in the manuscript, but in the submission system. | The source of funding is not presenting in the manuscript, but in the submission system. |

*Give information separately for cases and controls in case-control studies and, if applicable, for exposed and unexposed groups in cohort and cross-sectional studies.

**Note:** An Explanation and Elaboration article discusses each checklist item and gives methodological background and published examples of transparent reporting. The STROBE checklist is best used in conjunction with this article (freely available on the Web sites of PLoS Medicine at http://www.plosmedicine.org/, Annals of Internal Medicine at http://www.annals.org/, and Epidemiology at http://www.epidem.com/). Information on the STROBE Initiative is available at www.strobe-statement.org.
