# Supplementary material for: Association of Processed Meat Intake with Hypertension Risk in Hemodialysis Patients: A Cross-Sectional Study
Source: PLoS One. 2015 Oct 30;10(10):e0141917. doi: 10.1371/journal.pone.0141917 (PMC4627724; doi:10.1371/journal.pone.0141917)
Supplement: S1 Table — (DOCX) [file pone.0141917.s003.docx]

**S1 Table. Comparison of mean daily intake between two 3-day dietary records (Mean ± SE)**

|  | Crude data | | Energy adjusted data | |
| --- | --- | --- | --- | --- |
|  | First 3-day dietary record | Second 3-day dietary record | First 3-day dietary record | Second 3-day dietary record |
| Energy (kcal) | 1745.0 ± 51.2 | 1648.4 ± 145.1 | - | - |
| Protein (g/kg) | 1.0 ± 0.1 | 1.1 ± 0.1 | 0.7 ± 0.0 | 0.7 ± 0.1 |
| Sodium (mg) | 1576.5 ± 262.3 | 1591.3 ± 296.1 | 878.7 ± 73.6 | 917.8 ± 125.0 |
| Phosphate (mg) | 747.6 ± 51.6 | 701.6 ± 51.4 | 484.0 ± 18.0 | 428.5 ± 19.9 |
| Red meats (servings) | 1.9 ± 0.4 | 1.8 ± 0.4 | 1.2 ± 0.2 | 1.0 ± 0.2 |
| Processed meats (servings) | 1.2 ± 0.1 | 1.2 ± 0.1 | 0.8 ± 0.0 | 0.7 ± 0.0 |
| White meats (servings) | 2.0 ± 0.4 | 1.5 ± 0.3 | 1.4 ± 0.1 | 1.2 ± 0.1 |
| Soybeans (servings) | 0.7 ± 0.5 | 0.5 ± 0.2 | 0.6 ± 0.2 | 0.3 ± 0.1 |

*Significant difference from the first 3-day dietary record by *t*-test (normal distribution) or simple linear rank test (not normal distribution) (p < 0.05)
